# Plant Xyloglucan Xyloglucosyl Transferases and the Cell Wall Structure: Subtle but Significant

**DOI:** 10.3390/molecules25235619

**Published:** 2020-11-29

**Authors:** Barbora Stratilová, Stanislav Kozmon, Eva Stratilová, Maria Hrmova

**Affiliations:** 1 Institute of Chemistry, Centre for Glycomics, Slovak Academy of Sciences, Dúbravská cesta 9, SK-84538 Bratislava, Slovakia; barbora.stratilova@gmail.com (B.S.); chemsksa@savba.sk (S.K.); eva.stratilova@savba.sk (E.S.); 2 Faculty of Natural Sciences, Department of Physical and Theoretical Chemistry, Comenius University, Mlynská Dolina, SK-84215 Bratislava, Slovakia; 3 School of Life Science, Huaiyin Normal University, Huai’an 223300, China; 4 School of Agriculture, Food and Wine, University of Adelaide, Glen Osmond, SA 5064, Australia

**Keywords:** enzyme structure and function, GH16 family, homo- and hetero-transglycosylation reactions, molecular modelling and simulations, plant cell walls loosening and re-modelling

## Abstract

Plant xyloglucan xyloglucosyl transferases or xyloglucan endo-transglycosylases (XET; EC 2.4.1.207) catalogued in the glycoside hydrolase family 16 constitute cell wall-modifying enzymes that play a fundamental role in the cell wall expansion and re-modelling. Over the past thirty years, it has been established that XET enzymes catalyse homo-transglycosylation reactions with xyloglucan (XG)-derived substrates and hetero-transglycosylation reactions with neutral and charged donor and acceptor substrates other than XG-derived. This broad specificity in XET isoforms is credited to a high degree of structural and catalytic plasticity that has evolved ubiquitously in algal, moss, fern, basic Angiosperm, monocot, and eudicot enzymes. These XET isoforms constitute gene families that are differentially expressed in tissues in time- and space-dependent manners during plant growth and development, and in response to biotic and abiotic stresses. Here, we discuss the current state of knowledge of broad specific plant XET enzymes and how their inherently carbohydrate-based transglycosylation reactions tightly link with structural diversity that underlies the complexity of plant cell walls and their mechanics. Based on this knowledge, we conclude that multi- or poly-specific XET enzymes are widespread in plants to allow for modifications of the cell wall structure in muro, a feature that implements the multifaceted roles in plant cells.

**Table of Contents**

1. Plant cell walls and structure, and key components
2. Roles of xyloglucan xyloglucosyl transferases in cell wall formation and re-modelling
  Catalysis, and remarks on nomenclature and classification
  Enzyme activity assay methods
3. Substrate specificity of plant xyloglucan xyloglucosyl transferases
  Homo-transglycosylation reactions
  Hetero-transglycosylation reactions with neutral donor and acceptor substrates
  Hetero-transglycosylation reactions with charged (ionic) acceptor substrates
4. Implications of transglycosylation reactions catalysed by xyloglucan xyloglucosyl transferases in the cell wall structure, function and dynamics

## 1. Plant Cell Walls and Structure and Key Components

The presence of polysaccharide-rich cell walls (CWs) is a characteristic feature of plants and fungi. Throughout the evolution of plants, CWs have conformed to multiple roles, including mechanical support, diffusion and growth regulation, defence against biotic and abiotic stresses, and cell-to-cell communication. CWs are highly complex structural entities largely composed of organic polymeric molecules interlinked by covalent and non-covalent linkages. Properties of CWs depend on the composition and chemical linkages of individual adjoined components, and their structure [1,2,3,4,5,6].

**Figure 1:**
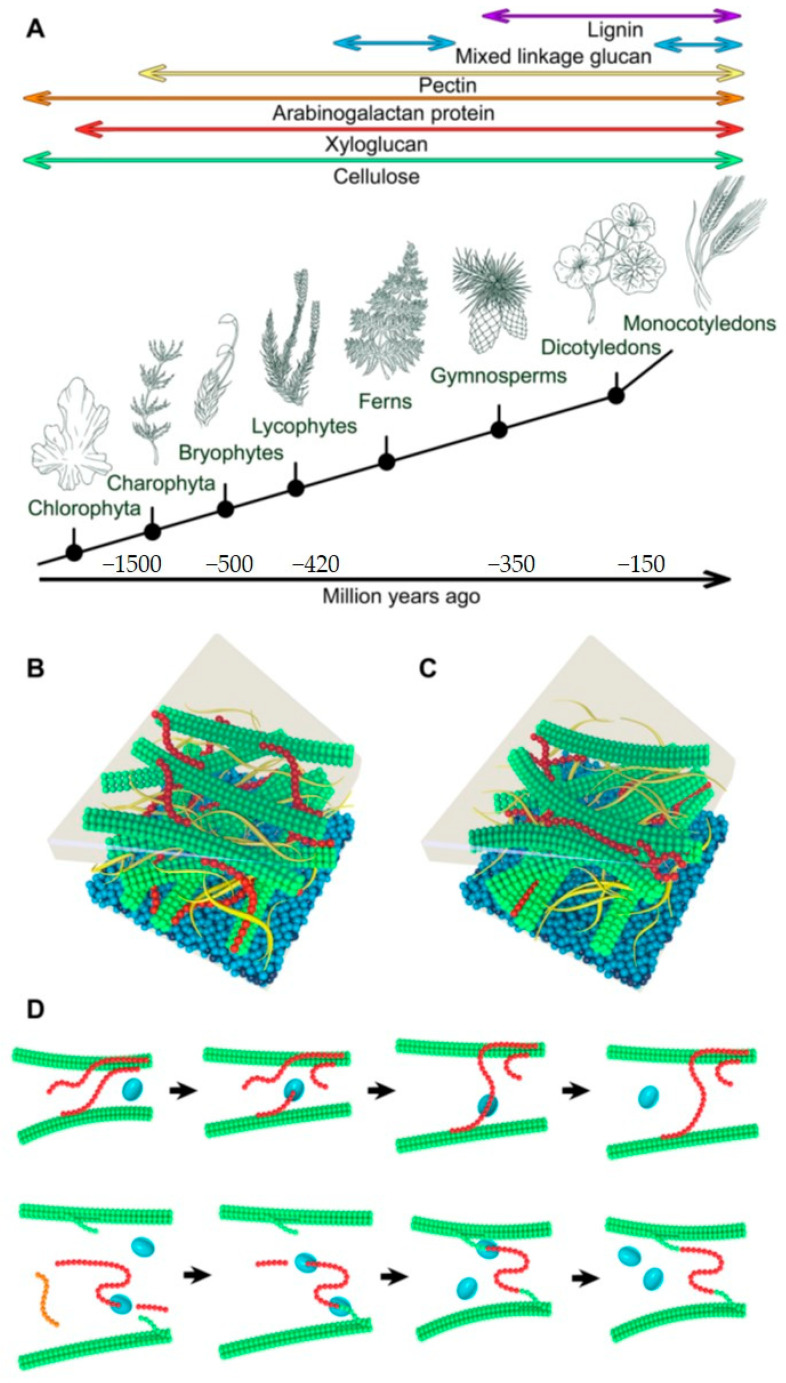
Polysaccharides form the structural foundation of plant CWs. (**A**) distinct polysaccharides emerge in plant CWs in phyla during the evolutionary history of plants [22,55]; (**B**,**C**) intuitive CW structural models by Albersheim et al. [56] (**B**), and Park and Cosgrove [57] (**C**); (**D**) mechanisms of action of XET enzymes leading to the loosening of CWs through homo-transglycosylation reactions (top panel) [1], and cross-linking cellulose and XGs through hetero-transglycosylation reactions (bottom panel) that could enhance tighter packing of cellulose micro-fibrils and other polysaccharides [58]. Cellulose micro-fibrils are in green and XGs in red in (**B**–**D**); pectins are in yellow in (**B**,**C**); XETs are in cyan in (**D**); the plasma membrane is in blue in (**B**,**C**).

All land plants classified in *Embryophytes* evolved from *Charophytes* green algae (Figure 1A) contain CWs assumed to be one of the most decisive factors that allowed for terrestrialisation [7,8,9,10]. Despite certain common components, the composition of CWs varies in species [11,12] and tissues [13,14,15,16,17]. The structure of plant CWs could also be affected by growth conditions [18] and some common distributions of main structural polysaccharides are observed that depend on the evolutionary history of a plant including algae [10,19,20,21,22]. However, certain polysaccharides could have evolved independently several times [16], and this is observed in (1,3;1,4)-β-d-glucans (mix-linkage glucans, MLGs) in *Pteridophytes* (ferns, whisk ferns, horsetails) [23,24,25] and *Poales* (grasses) [26] (Figure 1A). CWs of grasses differ from those of other higher plants in a lower content of xyloglucans (XGs) and pectins but in a higher content of heteroxylans [13].

Plants are known to construct two types of CWs, termed primary cell walls (PCWs) and secondary cell walls (SCWs) that differ in composition, structure, and function. The dynamic PCW structures comprise of the networks of cellulose micro-fibrils tethered by cross-linking glycans [27] that are embedded in the matrix of pectin substances [28,29] and glycoproteins. Glycans or hemicelluloses include variously substituted XGs, which are the major form of cross-linking glycans in dicotyledonous plants, while xylan(s)—Xyl(s), arabinoxylan(s)—AraXyl(s), glucuronoarabinoxylan(s), mannan(s)—Man(s), galactomannan(s)—GalMan(s), glucomannan(s)—GlcMan(s) and galactoglucomannan(s)—GalGlcMan(s), and MLGs largely replace XGs in monocots and lower plants [1,30]. Pectins [31] composed of the homogalacturonan (HG) part are cross-linked via Ca^2+^ bridges that causes the gelling effect, while the ‘hairy’ regions of pectins are made of rhamnogalacturonan I (RG I) and rhamnogalacturonan II (RG II) [32,33,34,35]. Conversely, SCWs that are more typical for woody and vascular tissues after their growth ceases are more rigid compared to PCWs since they contain more of other cross-linking glycans than pectins, and are reinforced by lignin, a hydrophobic phenylpropanoid polymer [36,37,38].

Cellulose, as the most abundant organic compound on Earth [39], consists of the repeating glucopyranose moieties linked through (1,4)-β-d-linkages that form micro-fibrils tightly bound via hydrogen bonds—these supra-molecular structures form the backbone of PCWs [40]. Cellulose is found not only in CWs of green algae and higher plants but also in *Rhodophyta* (red algae), *Phaeophyceae* (brown algae), *Oomycetes* (fungus-like microorganisms), *Ameobozoa*, animals, and in some *Procaryotes* (*Cyanobacteria*) [22].

The biosynthesis of XGs [41] seems to be reserved for plants including certain green algae [42]. XG was not detected in red and brown algae [22], and this absence is also supported by the lack of XG modifying enzymes in these organisms [43]. XG consists of repeating (1,4)-β-d-linked glucopyranosyl moieties [44], which contain the C-6 carbon branching by α-d-xylopyranosyl residues. Xylosyl moieties could be further substituted by galactopyranosyl residues on C-2 carbons (β-d-Galp-(1,2)-α-d-Xylp) and the galactosyl moieties could carry the fucopyranosyl branching (α-l-Fucp-(1,2)-β-d-Galp-(1,2)-α-d-Xylp). In certain instances, the arabinopyranosyl [45,46] and galacturonate [47,48] substituents are found, which suggests that the structure of XG differs from plant to plant, but also between the parts of the same plant [49,50,51]. The XG backbone is synthesised by a XG:glucan synthase encoded by members of the C subfamily of *cellulose synthase-like (CSL)* genes [41,52], but only when both UDP-glucose and UDP-xylose are present [53,54], meaning that the activity of another enzyme XG:xylosyltransferase is required to produce XG [53]. Most of XG residues are substituted with d-Gal by XG:galactosyltransferases and further modified with l-fucose by XG:fucosyltransferases [53,54], although more work is required to clarify all aspect of XG biosynthesis. A recent study with the C subfamily CSL synthases and their genes found a quintuple mutant with disruptions in five C subfamily *CSL* genes that had no detectable XG, and did not display the significant alteration of gene expression at the whole genome level [41].

The similarity between the structure of XG and cellulose underlies the conformational homology of these polysaccharides, which results in their strong noncovalent associations [54]. Networks of cellulose and XGs were for a long time considered to support the structure of PCWs, which also trigger their flexibility and strength [59,60,61,62,63]. The most recognised PCW model of dicotyledons [56] is based on linear micro-fibrils of cellulose, each consisting of 32 cellulosic micro-fibrils inter-connected through hydrogen bonds. These para-crystalline micro-fibrils are interwoven and bridged to polymeric XGs. According to this model (Figure 1B), the function of pectin is attributed to a gelling material that pervades the space between the cellulosic and XG structures. The advances in the field of microscopic techniques allowed for the development of advanced CW models based on so-called hot spots of cellulose micro-fibrils, where these micro-fibrils composed of eight or sixteen cellulosic subunits [64], come to close contacts with each other (Figure 1C). These hot spots of cellulosic micro-fibrils are isolated from each other by a thin layer of XGs, which according to this model are isolated in PCWs [57]. Here, pectins serve as the filling material between the cellulosic and XGs structures, and take over the major role of the XGs, compared to the first model. Various pectins interact both with cellulose and XGs, thus ensuring the flexibility and strength of CWs [65]. The processes of hot spots formation remain unknown, although one possibility is that they are formed spontaneously during the cellulose deposition into CWs, or that they could be formed enzymatically [66].

While there are numerous studies focused on the role of XGs in CW modifications during plant growth and development, little information is available as to how XGs participate in the CW formation. Recent work focused on the regeneration of CWs in wild-type *Arabidopsis thaliana* and a double mutant *xxt1 xxt2* lacking any detectable XGs, suggested that the formation of cellulosic networks is XG-independent [67].

As already noted, CWs show a remarkable diversity that underlies the function of each cell, which is directly linked to the basic and subtle structure of polymers, their quantity, ratios, and underpins mutual interactions. The syntheses of CW polysaccharides occur due to the cooperative activities of prodigious numbers of biosynthetic glycosyl transferases (GTs) or synthases [41,68,69,70,71,72,73,74,75,76,77,78,79,80,81,82,83,84], localised mostly in the Golgi apparatus [85]. The products of these GTs and synthases are transported to CWs by secretory vesicles, although the exception includes the superfamilies of cellulose synthases [86,87,88,89,90] and callose synthases [91] locating in the plasma membrane [92]. In the latter instances, polysaccharides synthesised by these enzymes could be exported directly to CWs. However, the structural polysaccharides that are observed in CWs are often heterogeneous. This is assumed to be achieved through a wide range of available activated sugar donors required for GTs activities, which could give rise to a variety of glycosidic linkages using different sugar isomers [16].

Life processes in plants are underscored by CWs structure and re-organisation, which involves disintegration, elongation and expansion. In addition to GTs, these processes are governed by hydrolases and lyases, and non-catalytic expansin proteins [93,94,95,96]. As in the case of GTs, there is a vast number of enzymes that could modify structural polysaccharides in muro by cleaving bonds, esterifying or de-esterifying saccharide moieties [97,98], and incorporating new material into CWs or re-constructing CW polymers by cross-linking. The last two processes are secured by xyloglucan xyloglucosyl transferase also known as xyloglucan endo-transglycosylase (XET) enzymes (Figure 1D), which could either loosen or enhance the packing of cellulosic micro-fibrils and other polysaccharides in CWs [58,99].

Xyloglucan xyloglucosyl transferases or XET enzymes (EC 2.4.1.207) as one of the key glycosidic bond-formation enzymes participating in plant CW expansion, reconstruction, and re-modelling [100,101,102,103,104] were independently discovered by three groups [105,106,107]. According to the Enzyme Commission [108], these enzymes are also named as endo-xyloglucan transferases.

## 2. Roles of Xyloglucan Xyloglucosyl Transferases in Cell Wall Formation and Re-Modelling

*Catalysis, and remarks on the nomenclature and classification*—The nomenclature of xyloglucan xyloglucosyl transferases or XET enzymes is defined by the International Union of Biochemistry and Molecular Biology (IUBMB)/International Union of Pure and Applied Chemistry (IUPAC) Biochemical Nomenclature Committee [108] that is also implemented in the Kyoto Encyclopaedia of Genes and Genomes Enzyme Database (KEGG). These enzymes are classified amongst transferases by the Enzyme Commission (EC) and listed under the primary identifier EC 2.4.1.207 in IUBMB/IUPAC and the BRENDA collection of enzyme functional data [109]. The differences in the used nomenclature are based on whether the authors consider the transfer of ‘glycosyl’ (xyloglucan endo-transglycosylase) or ‘glucosyl’ (xyloglucan endo-transglucosylase) groups [110,111,112,113,114,115,116,117], although given that XETs primarily transfer XG fragments, the usage of the first name should be preferred. The fundamental feature of the catalysis mediated by XETs is the breaking of a bond between 1,4-β-d-linked glucosyl residues of XGs and the transfer of an XG fragment onto O-4 of the non-reducing terminal end of the glucose moiety of the acceptor, which can be XG or its oligosaccharide (XG-OS). This constitutes a so-called ping-pong bi bi reaction mechanism rather than a sequential one [118,119]. It is of note that the definition by the Enzyme Commission contains a strict note ‘does not use cello-oligosaccharides as either donor or acceptor’, although, in the light of the current knowledge, this specification is obsolete [58,99,120,121,122,123,124,125].

**Figure 2:**
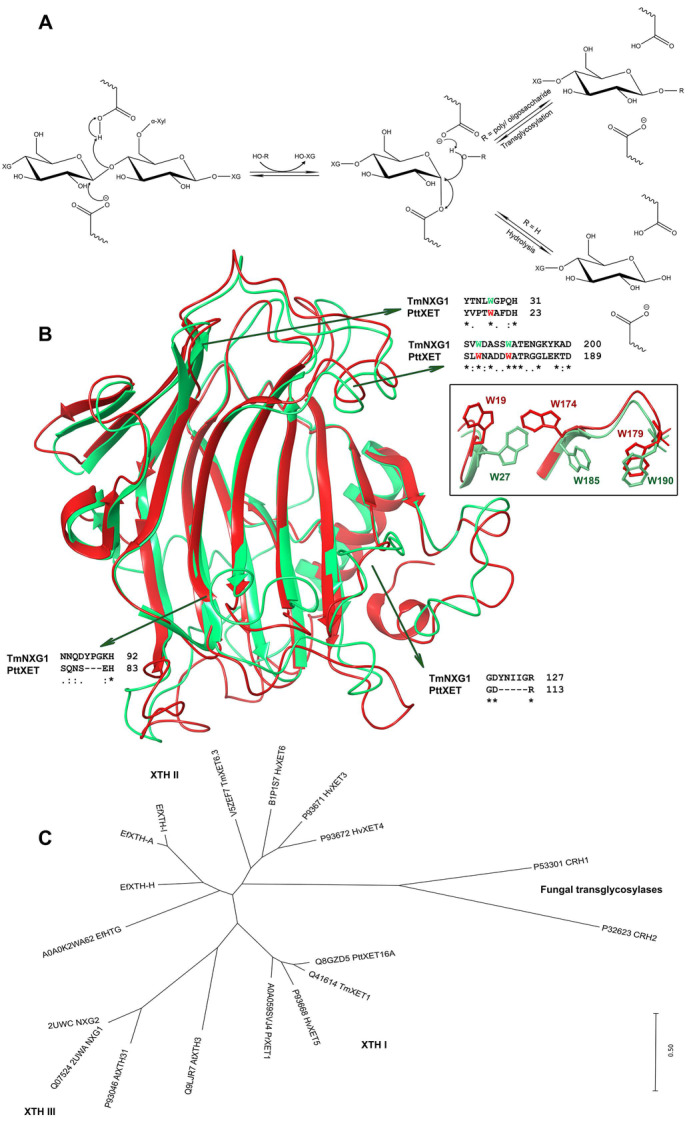
The GH16_20 subfamily of the XTH enzymes. (**A**) reaction mechanism [138] leading to transglycosylation or hydrolytic reactions; (**B**) superposition of the crystal structure of poplar PttXET16A transglycosylase (PDB accession 1UN1; red) and the nasturtium TmNXG1 hydrolase (green) [138,140] points to structural differences that underlie their distinct activities; differences in selected signatures that underlie these activities are indicated by sequence alignments, and some of these residues shown in the inset; (**C**) unrooted phylogenetic tree of the GH16 subfamily (MEGA v7.0.26; [141]) shows clustering of entries into three subgroups, where subgroups I and II consist of transglycosylases and subgroup III of hydrolases [121,123,125].

A more objective view on the XET enzyme nomenclature and classification is given by the Carbohydrate-Active enZYmes Database (CAZy; CAZypedia Consortium 2018) [126], which is based on protein tertiary structures and substrate specificities or activities. According to CAZy, XETs are classified in a glycoside hydrolase (GH16 family) and not in a glycoside transferase (GT) group. According to this classification, the latter group contains enzymes which utilise activated sugar donors. In accordance with tertiary structures of XET enzymes, the first steps of both transglycosylation and hydrolytic reactions are binding and cleavage of donor substrates. The difference occurs in the second step, in which the fragment with the original non-reducing end of the substrate is transferred to an acceptor, which in the case of a typical transglycosylase is another saccharide, while, in the case of a hydrolase, it is a water molecule (Figure 2A) [127]. This second step of the reaction has the key importance for the nomenclature and classification of transglycosylases as transferases. Commonly, most hydrolases could also transglycosylate, but this only occurs under high substrate concentrations [128], when in the later stages the products accumulate and shift the chemical equilibrium of the reaction towards transglycosylation reactions, due to a specific response of the biocatalyst. Contrary to this, ‘true’ transglycosylases, which include XETs, catalyse primarily the transfer on a saccharide from the beginning of the reaction. While hydrolytic reactions catalysed by hydrolases reflect the increased concentrations of reducing groups in the reaction system, this is not the case for ‘true’ transglycosylases. Furthermore, during reactions with endo-transglycosylases, no dramatic decrease in the viscosity of the polymeric substrates is observed at the early stages of reactions, contrary to endo-hydrolases. Besides XETs, other transglycosylases or enzymes with potential transglycosylase activities were described in plants as endo-transglycosylases/hydrolases recognising (1,4)-β-d-mannan-derived polysaccharides [129,130], xylan endo-transglycosylases functionalising heteroxylan polysaccharides [131,132,133], MLG: xyloglucan endotransglucosylases [134,135] recognising MLGs, and the hetero-trans-β-glucanase (HTG) that functionalise cellulose [96,136], although designating these enzymes as such hides the fact that the latter enzymes are broad specific or poly-specific XET enzymes.

XET enzymes are classified in GH family 16 (GH16) in CAZy. A GH16 family is a large group, which was according to specific features in tertiary structures further sub-divided into 23 subfamilies [137]. Subfamily GH16_20 includes XETs and xyloglucan endohydrolases (XEHs, EC 3.2.1.151) [138,139] with predominantly hydrolytic activities towards XGs. This group of enzymes contains the products of *XTH* (xyloglucan transglycosylase/hydrolase) genes encoding both types of XG-modifying enzymes which display close similarity in their tertiary structures [140].

**Figure 3:**
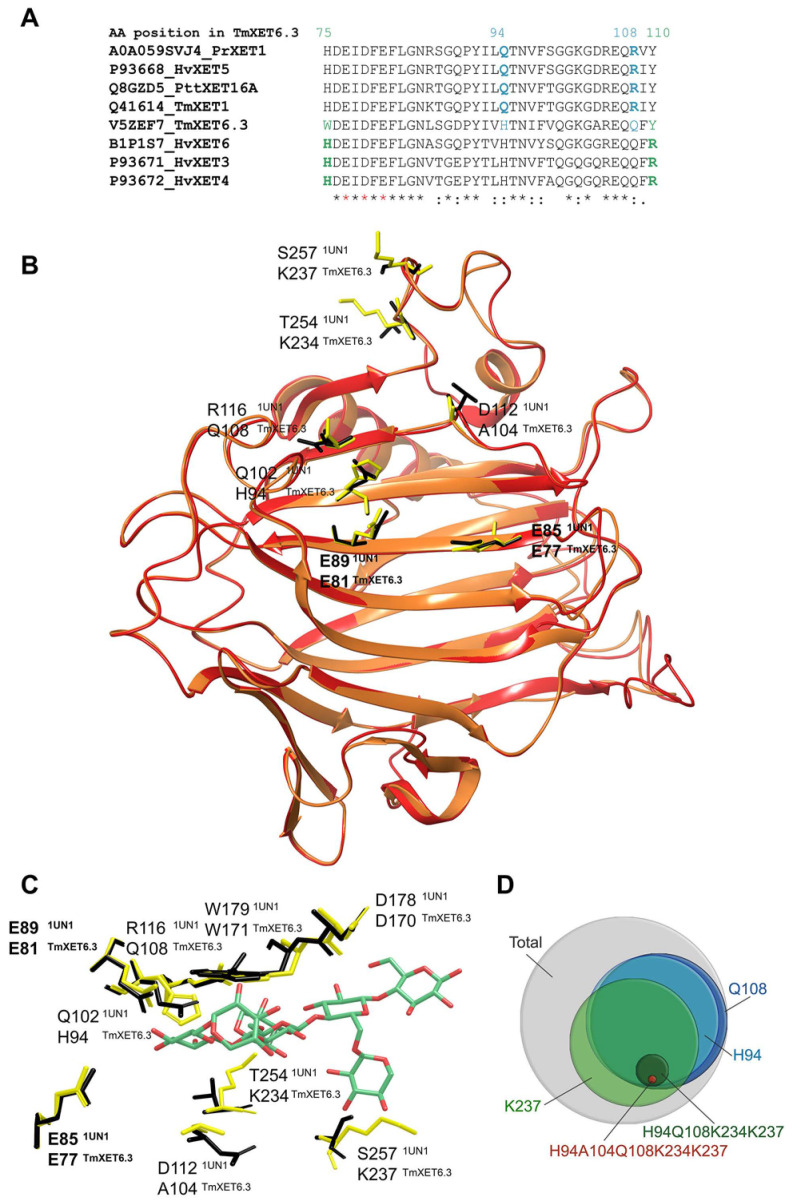
Structural basis of hetero-transglycosylation reactions catalysed by plant XETs with neutral acceptor substrates. (**A**) sequence alignment of specific and nonspecific XETs to indicate target residues destined for mutagenesis in TmXET6.3 to suppress the hetero-transglycosylation activity with neutral acceptors (blue, mutations H94Q and Q108R) and evoke the hetero-transglycosylation activity with the charged [α(1-4)GalA*p*]_5_ acceptor (green, mutations W75H, Y110R). Red asterisks mark catalytic residues [125]; (**B**) superposition of poplar PttXET16A (PDB accession 1UN1; red) and the TmXET6.3 model [123] orange; points to differences in residues between the two structures, which are visualised in yellow (PttXET16A) and black (TmXET6;3) sticks; (**C**) interactions of the XXXG acceptor (cpk green) with the residues of PttXET16A (yellow sticks) and TmXET6.3 (black sticks); (**D**) Venn diagram of the occurrence of nonspecific XETs (with detailed residue configurations) based on the analysis of 3394 UniProtKB entries [123].

**Figure 4:**
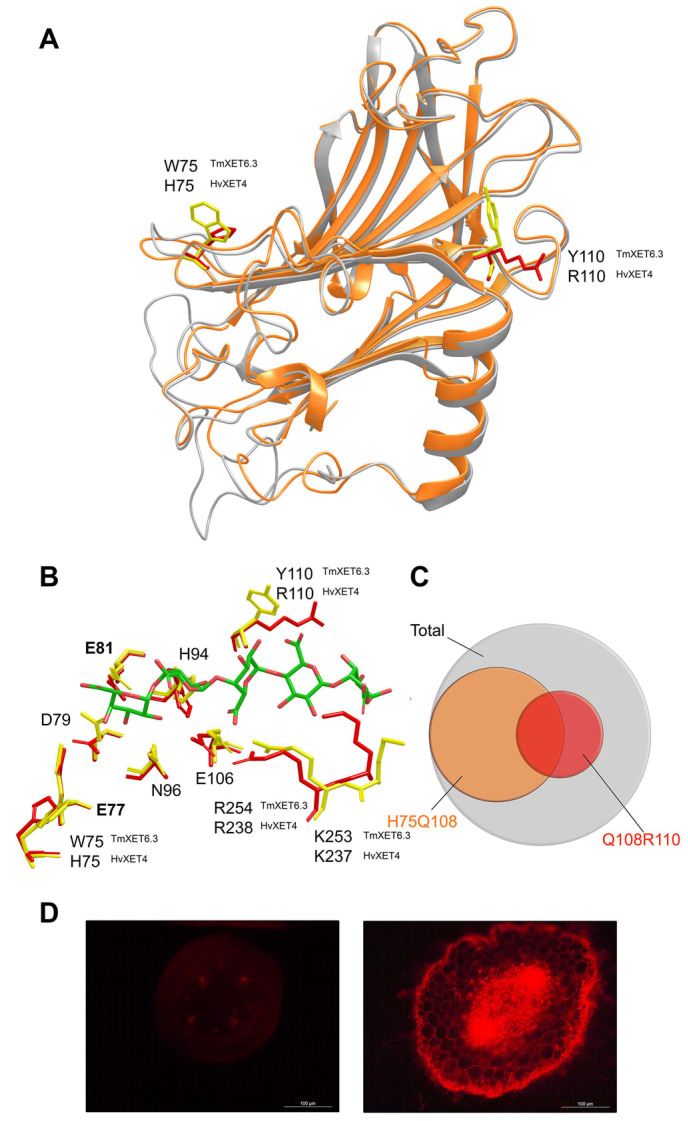
Structural basis of hetero-transglycosylation reactions catalysed by plant XETs with the negatively charged homogalacturonan [α(1-4)GalA*p*]_5_ acceptor substrate. (**A**) 3D models of TmXET6.3 (orange) and HvXET4 (gray) indicate target amino acid residues destined for mutagenesis in TmXET6.3, to evoke the binding of the [α(1-4)GalA*p*]_5_ acceptor and catalyse hetero-transglycosylation reactions with XG or HEC as donors, similarly to HvXET4 [125]; (**B**) interactions of [α(1-4)GalA*p*]_5_ (cpk green) with the residues of HvXET4 (yellow sticks), where the corresponding residues in TmXET6.3 are indicated in red sticks; (**C**) Venn diagram of the occurrence of nonspecific XETs (with detailed residue configurations) with the potential to link XG or cellulose to ([α(1-4)GalA*p*]_5_) based on the analysis of 3394 UniProtKB entries [125]; (**D**) incorporation of fluorescently labelled [α(1-4)GalA*p*]_5_ into the stem (left) or root (right) cells of the nasturtium seedling. The pronounced labelling of root epidermal and vascular bundle CWs are visible, while no or little incorporation of [α(1-4)GalA*p*]_5_ is seen in stem cells. Labelling was performed as described [125]. Scale-bar lengths are indicated.

Currently, the best characterised XET enzyme is the PttXET16A isoform from hybrid aspen, *Populus tremulus x tremuloides* [138,140]. After its expression in a recombinant host achieved in high yields in *Pichia* [102], the crystal structure of PttXET16A [127] (Protein Data Bank—PDB accessions 1UN1 and 1UMZ) revealed that the enzyme folds into two antiparallel β-sheets, which form a β-sandwich consisting of convex and concave regions. The catalytic machinery, formed by two glutamic acid residues Glu85 and Glu89, with an aspartate Asp87, is located approximately mid-way in the convex region. The C-terminal end is elongated compared to other XTH family members [138,140] and located near the convex region of the β-sheet, forming an α-helix and another β-strand on the concave side of the molecule; this part of the structure is stabilised by two disulphide bridges. PttXET16A is N-glycosylated at Asn93 with two N-acetylglucosaminyl and mannosyl moieties that are stabilised by hydrogen bonds. The structures of other plant XET enzymes are yet to be determined, however, the structural features of barley and nasturtium XETs (Figure 2B, Figure 3B, and Figure 4B) and HTG were defined through homology modelling, an approach that could introduce local approximations in structural features of modelled XETs [121,123,125,136,142].

The mechanism of transglycosylation catalysed by XET enzymes proceeds in two stages that incorporate two transition states (Figure 2A). The first step is the deprotonation of the carboxyl acid residue acting as the nucleophile that attacks the anomeric carbon forming the glycosyl-enzyme intermediate complex with acidic assistance provided by the acidic carboxylate. In PttXET16A, the nucleophile attacking the anomeric carbon is Glu85, while Glu89 acts as an acid/base, which protonates the released saccharide and subsequently de-protonates the glycosyl acceptor. The Asp87 residue located mid-way between both catalysts Glu85 and Glu89 controls the protonation state of the catalytic machinery and operates through hydrogen bonding interactions. The nucleophile must be de-protonated during the donor substrate attack, while Asp87 and Glu89 are protonated and donate the proton to a leaving saccharide. During the later stages of the transfer reaction, the glycosyl-enzyme intermediate complex dissociates after the nucleophile attack on the anomeric carbon, and a new glycosidic bond is formed [127,138].

Interactions between residues in PttXET16A and dimeric XG nonasaccharide were obtained using molecular dynamics simulations [140]. The substrate was modelled in active site in a way, in which one of the XG nonasaccharide dimers occupied the donor site creating a stable intermediate with the enzyme, while the second XG nonasaccharide occupied the acceptor site. It was important to observe that the XG nonasaccharide of the reducing-end glucose moiety changed its conformation into a boat at the beginning of the simulation and kept this conformation during the whole simulation time. This was the case not only for PttXET16A, but also for XEHs [140].

Attention was paid to structural differences, which determine whether the XET/XEH enzymes act as transglycosylases (PDB accessions 1UN1, 1UMZ) or hydrolases (TmNXG1; PDB accession 2UWA) [138,139,140,143]; here, the information of their primary and crystal structures was compared including the TmNXG1-DELTAYNIIG mutant (PDB accession 2VH9). Minor differences in structures lead to stronger binding of a donor substrate combined with larger loop flexibility at the acceptor binding site, and the higher conformational flexibility of specific residues underlined the hydrolytic preference [140,143] (Figure 2B). This was also reflected by the phylogenetic analyses, which showed that the distribution of XTH gene products was segregated into three groups, with XEHs belonging to the XTH III clade, while XETs were placed in both XTH I and XTH II clades (Figure 2C, Supplementary Data Set S1) [121,123,138,144]. Based on these structural and phylogeny insights, it was postulated that GH16 hydrolases could have evolved from XET transglycosylases [138,143], although the research including a gradual cross-genome survey and the whole GH16 family phylogeny continues [125,145].

*Enzyme activity assays methods*—Preferred methods of XET activity assays are based on the use of radiochemically or fluorescently labelled acceptor oligosaccharides and an unlabelled donor polysaccharide. Alternatively, radiolabelled donor and unlabelled acceptor substrates could be used [96]. Products then contain labelled components that are incorporated into products, and remaining unincorporated donors or acceptors are removed through washing on a filter-paper [146], through a gel [120,147], size-exclusion chromatography [58,107,123], or ethanol precipitation [148]. Labelled oligosaccharides could also be used in high-throughput activity assays [149] and for visualisations of XET activities in vivo [150,151,152,153]. Despite an undeniable advantage of the latter technique for screening of XET activities, it is important to consider ongoing reactions amongst polysaccharides in muro. For the selection of activity assays, it is crucial to consider the choice of fluorescent tags [154,155] and the removal of unreacted donors or acceptors [99,123]. Other XET activity assays include viscometry and colorimetry [156].

## 3. Substrate Specificity of Plant Xyloglucan Xyloglucosyl Transferases

Two major subtypes of reactions catalysed by XET enzymes are known that reflect the chemical nature of substrates and structural characteristics of enzymes that recognise these saccharide substrates. These reactions are termed homo-transglycosylation reactions with XG-derived donors and acceptors, and hetero-transglycosylation reactions that utilise neutral donor and neutral or ionic acceptor saccharides other than XG-derived. The concise summary of characterised XET enzymes and their isoforms is summarised in Table 1.

*Homo-transglycosylation reactions with XG-derived donor and acceptor substrates—*The PttXET16A enzyme from hybrid aspen *Populus tremulus x tremuloides* (EC 2.4.1.207; defined by the IUBMB/IUPAC Biochemical Nomenclature Committee) is considered to be an XET enzyme that utilises XG-derived donors and acceptors, meaning that it is XG-specific with a narrow substrate specificity [127,138]. A similar specificity was demonstrated in *Pinus radiata* PrXTH1 using computational approaches [157]. Both enzymes belong to the I/II cluster of *XTH* gene products [138,157]. Hetero-transglycosylation activities in PttXET16A were not detected with cellooligosaccharides (Cello-OS) [138], although in silico data with PrXTH1 indicated the weak binding of cellooctaose [157]. Based on evolutionary analyses of the GH16 family, spanning monocots, eudicots, and a basal Angiosperm [125], the PttXET16A enzyme clustered in the proximity of the barley HvXET5 isoform that catalysed these reactions at high rates [58].

*Hetero-transglycosylation reactions with neutral donor and acceptor substrates—*The first evidence for the existence of XETs that catalysed hetero-transglycosylation reactions, i.e., to mediate the transfer between structurally distinct saccharides, comes from the work of Ait Mohand and Farkaš [120]. These authors described the glycosyl transfer from XG to Cello-OS, laminarioligosaccharides (La-OS), and from the soluble carboxymethyl cellulose (CMC) and HEC cellulose derivates to XG-OS. These enzyme activities were detected in crude protein extracts prepared from germinating nasturtium seeds in polyacrylamide gel after isoelectric focussing [147]. Before this evidence, the substrate promiscuity in XETs from *Poaceae* was predicted based on molecular modelling of the GH16 family [158].

Hetero-transglycosylation activities of XETs with Cello-OS and cellulose conflict with the IUBMB/IUPAC Biochemical Nomenclature and Classification System [108], and recent research of these enzymes complicates this situation further. The key question arises if enzymes catalysing hetero-transglycosylating reactions could still be classified as XETs and, if so, what is the allowable limit for the ratio of the activity with the XG/XG-OS substrate pair and structurally different substrates. Another problem arises from the fact that currently the substrate specificity of only a few XETs in near-homogenous forms was defined, and therefore a given substrate specificity could unequivocally be assigned to a handful of XET enzymes. The latter criterion of near-homogeneity has not been always followed, whereby undefined crude protein extracts were used for substrate specificity definitions of XET enzymes.

The first XET in a near-homogenous form with a defined primary structure catalysing hetero-transglycosylation reactions was the HvXET5 isoform from barley (*Hordeum vulgare* L.) [58]. Except for XG and XG-OS, this enzyme in vitro mediated the formation of covalent linkages between XGs, CMC, HEC, and MLGs (donor substrates) and XG-OS or Cello-OS (acceptor substrates). The efficiency of the covalent bond formation with HEC was comparable to that with XG (44%), whereas it was lower but significant with MLGs (0.2%), and the formation of the hybrid saccharide products was defined by mass spectrometry. The substrate specificity was also defined for another near-homogenous barley HvXET6 isoform, where the efficiencies of transfer between XG and Cello-OS with degrees of polymerisation (DPs 2–6) for HvXET5 and HvXET6 were similar [121].

HEC and Cello-OS substrates functioned as respective donors and acceptors also for partially purified XET isoforms from parsley roots (*Petroselinum crispum*) [148]. The same was observed for XETs isolated from parsley stems and leaves, and from stems, leaves, and roots from nasturtium [123]. The efficiencies of these activities did not exceed 5% for those with HEC as the donor, and 0.5% for Cello-OS as an acceptor compared to homo-transglycosylation reactions with the XG/XG-OS pair. In comparison, 23% efficiency was reported for a crude nasturtium protein extract from germinating seeds with the HEC/XG-OS substrate pair [120].

Other XET enzyme recognising cellulose as the donor substrate is AtXTH3 from *Arabidopsis thaliana*, which in near-homogenous form catalysed hetero-transglycosylation reactions between cellulose and Cello-OS, cellulose and XG-OS, in addition to the homo-transglycosylation reaction with the XG/XG-OS pair, which was the dominant reaction. Notably, AtXTH3 also produced cello-oligomers with higher DPs than the original aminopyridyl cellohexaose substrate, characterised by mass spectrometry, and insoluble cellulose-like material in the absence of other substrates [122]. Despite differences in the ability to bind cellulose-derived substrates and the yields of transglycosylation products, HvXET5 [58] and AtXTH3 [122] clustered within the same phylogenetic group of XTH I, as presumably XG-specific PttXET16-34 and PrXTH1 [138,157], although they segregated to different sub-clades (Figure 2C).

Further XETs with known primary structures and the ability to utilise besides XG also cellulose or MLGs as donors, are three acidic EfXTH-A, EfXTH-H, and EfXTH-I isoforms from *Equisetum fluviatile* [117], although the homogeneity of these enzymes was not demonstrated. Homo-transglycosylation activity with the XG/XG-OS pair was the dominant reaction for all isoforms and the efficiencies with cellulose and MLGs varied. EfXTH-A displayed the comparable transfer of MLG fragments to XG-OS, as was the case of HvXET5 (0.2–0.3%) [58], while the activity with cellulose was incomparably higher with HvXET5. EfXTH-H and EfXTH-I showed equivalent hetero-transglycosylation activities with both MLGs and cellulose donors that were around an order of magnitude higher than that of EfXTH-A [117]. Conversely, HTG and MLG: xyloglucan endotransglycosylase from *Equisetum* preferred cellulose and MLGs as donors with XG-OS [117]. Such enzymes could only be found in various *Equisetum* species and charophytic algae [134], and their predicted function is to re-model hemicelluloses in horsetails shoots but not in a horsetail callus [135]. HTG belongs to the GH16_20 subfamily [137] and was characterised at the structural level [136]. 

Homology modelling of HTG suggested that three amino acid residues Pro10, Ser34, and Leu245 were responsible for the evolution of their substrate specificity and that Pro10 and Ser34 participated in the binding of the donor and Leu245 in the binding of the acceptor substrates [136]. It is notable that, in other XETs, Pro10 is substituted by Trp and Ser34 by Gly [117] and that enzymes with high reaction rates with cellulose donors, such as HvXET5 [58] and AtXTH3 [122] also contain P10W and S34G mutations.

It was further suggested that, in HTG, the R246L mutation could underlie differences in the binding of Cello-OS [136], although TmXET6.3 [123], EfXTHs [117], and HvXET3, HvXET4 and HvXET6 [125] that also bind Cello-OS, have Arg in equivalent positions, whilst AtXTH3 [122] contains the Lys residue.

The broader acceptor substrate specificity of XETs from nasturtium germinating seeds [120] initiated in-depth studies of their isoforms [159] and the development of suitable methods for the detection of hetero-transglycosylation activities [123,149]. Additional activities of nasturtium XET isoforms with the XG-OS, Cello-OS, and La-OS acceptors [120] were defined with the oligosaccharides derived from MLG (MLG-OS), xylan (Xyl-OS), glucomannan (GlcMan-OS), galactomannan (GalMan-OS), and surprisingly with pustulan (Pu-OS) [123,149], which is found mostly in fungal CWs [3]. However, the data showed that the incorporation of Pu-OS in vivo was directed to the plasma membrane rather than to nasturtium CWs [160], but that this activity was also mediated by recombinant TmXET6.3 that locates to germinating seeds [123]. This TmXET6.3 was solely responsible for all additionally found hetero-transglycosylation activities [159]. Some of these activities were found in parsley and comparably to nasturtium they located to germinating seeds [123]. Crude protein extracts from nasturtium germinating seeds also catalysed hetero-transglycosylation reactions with XGs and HEC [120] and glucuronoxylan and GalMan [149]. However, besides XG and HEC, recombinant TmXET6.3 in a near-homogenous form could not utilise the above-listed donor polysaccharides [123], suggesting that this broad substrate specificity detected in crude protein extracts could belong to other nasturtium XET isoforms; this observation further emphasises the need for using near-homogenous XETs to unequivocally define substrate specificity.

TmXET6.3 belongs to the group of XTHs II (Figure 2C, Supplementary Data Set S1), and in its recombinant and a near-homogenous form it displayed the hetero-transglycosylation activities with neutral acceptors, such as those derived from arabinoxylan (AraXyl-OS), arabinan (Ara-OS), arabinogalactan (AraGal-OS), mannan (Man-OS), glucomannan (GlcMan-OS), and galactomannans (GalMan-OS) in combination with donor XG or HEC [123]. Reaction rates differed significantly in the following order: MLG-OS > Cello-OS > Pu-OS > AraXyl-OS > La-OS > Xyl-OS GlcMan-OS > Ara-OS, where tiny activities were observed with AraGal-OS, Man-OS, and GalMan-OS, and negatively charged oligogalacturonates did not serve as acceptors. Other factors that affected TmXET6.3 activities were DPs of Cello-OS acceptors and for MLGs the positions of (1,3)-β or (1,4)-β bonds [123].

Comparative bioinformatics of TmXET1 [161], PttXET16A [127], HvXET5 [58], PrXTH1 [157], and TmXET6.3 [123] (Figure 3A), including structural analyses of TmXET6.3 suggested that the efficiencies of hetero-transglycosylation reactions of TmXET6.3 could be modified via a single (H94Q, Q108R, K237T) or multiple mutations (H94Q/A104D/Q108R, K234T/K237T) (Figure 3B). The key residue that underlined this activity in TmXET6.3 was Q108 that interacted with saccharide moieties near the catalytic site, while K237, located at the additional β-sheet of the binding site sited near the C-terminus, restrained the acceptor to the position crucial for the reaction (Figure 3C). The effects of single Q108R and K237T mutations were less effective than when both were implemented together. The only exception in the observed suppression of hetero-transglycosylating activities were the reactions with Cello-OS that were significantly increased [123]. These mutational analyses inspired the large-scale bioinformatics analyses of residue positions in available UniProtKB XET entries (3394). Here, the presence of H94, Q108, and K237 occurs in 1%, 4%, and 10% respective cases, H94/Q108 in 11% cases, H94/Q108/K237 in 25% instances, H94/Q108/K234/K237 in 2% cases, and H94/A104/Q108/K234/K237 in 0.1% instances (Figure 3D). It was concluded that the enzymes with a predisposition to catalyse hetero-transglycosylation reactions could be more widespread in plants than previously thought.

Comparisons of primary structures of TmXET6.3 and barley HvXET3, HvXET4 [142,154] and HvXET6 [121,142] isoforms (Figure 3A) indicated that all barley enzymes should transfer fragments of XG or HEC to the same wide spectrum of oligosaccharides as it was found in TmXET6.3 because they contained suitable residues H94, Q108, and except for HvXET6 also K237 in positions crucial for the acceptor substrate specificity. This comparison showed that a full grasp of the structural basis of hetero-transglycosylation required the examinations of roles of lysine residues disposed around Arg238 (numbering of TmXET6.3). Further assessments of activities confirmed the assumptions on the hetero-transglycosylation activities of HvXETs towards neutral acceptors. However, these assessments also led to the discovery of hetero-transglycosylation activities with negatively charged oligosaccharide acceptors that have never been observed before [125].

*Hetero-transglycosylation reactions with a charged (ionic) acceptor substrate*—These activities were investigated with recombinant HvXET isoenzymes in near-homogenous forms [121,144,154]. As mentioned above, all tested neutral oligosaccharides served as convenient acceptor substrates for HvXET3, HvXET4, and HvXE6 isoforms although hetero-transglycosylation efficiencies differed between isoforms [125]. Unlike TmXET6.3, both HvXET3 and HvXET4 could transfer fragments of XG or HEC to the penta-galacturonic acid ([α(1-4)GalA*p*]_5_), which represents a fragment of the linear part of pectin, known as homogalacturonan. While the highest hetero-transglycosylation activity in HvXET3 was observed with the XG/[α(1-4)GalA*p*]_5_ substrate pair, the highest activity with the HEC donor was observed with Cello-OS. HvXET6 could also transfer XG fragments to [α(1-4)GalA*p*]_5_ but with lower efficiency, whilst Cello-OS were the preferred acceptors with the XG donor [125]. These findings were confirmed by homology modelling of barley isoforms and suggested that their substrate promiscuity resulted from minor changes in protein sequences and thus structural arrangements [123,125]. The molecular models of HvXET3 and HvXET4 superposed with TmXET6.3 (Figure 4A) and containing docked XG heptasaccharide (XXXG) donor and [α(1-4)GalA*p*]_5_ acceptor substrates at the −4 to +5 subsites pointed to those critical interactions between residues and saccharide moieties of [α(1-4)GalA*p*]_5_ (Figure 4B) [125]. To evaluate the statistical distribution of XETs with the potential to catalyse hetero-transglycosylation reactions using the XG/[α(1-4)GalA*p*]_5_ substrate pair, a total of 3394 UniProtKB XET entries were examined, where about 44% of XETs were identified to be able to do so (Figure 4C) [125]. Finally, the activities of hetero-transglycosylating XETs [153] were detected in vivo in CWs of barley roots where they incorporated fluorescently-labelled [α(1-4)GalA*p*]_5_) [125]. The latter activity was also detected in nasturtium but solely in roots and not stems (Figure 4D).

The information on enzyme–substrate interactions combined with comparative sequence analyses of barley isoforms and TmXET6.3 (Figure 3A) allowed the engineering of mutants in TmXET6.3 that could catalyse hetero-transglycosylation reaction with the XG/[α(1-4)GalA*p*]_5_ substrate pair, while wild-type TmXET6.3 lacked this activity. Single mutations of W75H and Y110R led to the binding of this [α(1-4)GalA*p*]_5_ ionic acceptor and the implementation of the double mutation W75H/Y110R increased this activity. Although the XG-specific enzymes have in the position corresponding to 75 in TmXET6.3 the His residue (Figure 3A), these enzymes could not utilise the [α(1-4)GalA*p*]_5_ acceptor due to the lack of Q108 that is also required for the binding of different oligo- or polysaccharides. As it is the case of hetero-transglycosylation reactions with neutral acceptors, the detailed examination of the function of lysine residues near Arg238 could bring new information on acceptor binding in TmXET6.3 and in barley isoforms.

In summary, it seems that there are only a few key residues in XETs, a combination of which leads to particular substrate specificity, and a certain homo- and hetero-transglycosylation ratio that underpins the utilisation of a variety of individual donor and acceptor substrates. Despite the unquestionable success of identifying these residues in XETs [123,125], it is imperative to solve more of the tertiary structures of these enzymes, in addition to unravelling more of XET protein sequences from a variety of organisms. It would be useful to focus on XETs, which exert a range of substrate specificities, e.g., on barley and *Equisetum* isoforms, as this information could contribute to the progress in the XTH field.

## 4. Implications of Transglycosylation Reactions Catalysed by Xyloglucan Xyloglucosyl Transferases in the Cell Wall Structure, Function and Dynamics

From the phylogeny point of view, it seems that the first XET enzymes have appeared along with XGs in CWs of green algae and were not found in red or brown algae [22,43,162]. However, it is not possible to establish whether other transglycosylases with related substrate specificities, e.g., recognising cellulose as the donor substrate, existed before the emergence of XET enzymes in red or brown algae, as it is the case of HTG that functionalises cellulose [136]; exploring these evolutionary events would open exciting avenues in the XET field.

The expression of *XTH* genes is independently regulated by various factors, e.g., hormones and environmental changes. As a consequence, XETs evolved in multiple isoforms, which fulfill various roles during different stages of plant growth and development and in response to biotic and abiotic stresses [111,114,159,163,164,165,166,167,168,169,170,171]. Elevated expression of *XTH* genes can also be observed during fruit ripening that leads to CW loosening but also stiffening, thus allowing for these CWs to be conditioned for future modifications and degradation by other enzymes [172,173]. *XTH* genes further play an irreplaceable role in saccharide metabolism connected to plant growth since their elevated expression during cell elongation affects properties of CWs that underlie sizes and shapes of plant tissues [104]. It is also assumed that the *XTH* gene products play key roles in the re-structuralisation of CWs [102]. Tissue localisation and suggested function of selected XET, XTH and other GH16 family enzymes examined in this work are defined in Supplementary Data Set S1.

As it was mentioned before, XETs are involved in the depolymerisation of plant structural polysaccharides such as XGs and cellulose, and in the incorporation of newly synthetised components within these polymers and their cross-linking [99,100,101,127,174,175,176,177]. XET enzymes primarily work on XG molecules associated with cellulose micro-fibrils in a way that does not affect the integrity of CWs [1,58] (Figure 1D). The discovery of XET isoforms that link effectively not only XG molecules but also those between XGs and cellulose or between celluloses themselves, such as HvXET5 [58] and AtXTH3 [122], or between XG or MLG and cellulose catalysed by HvXET5 [58] and HTG [96,124,136,177], or between XG and a whole spectrum of structurally distinct neutral saccharides such as TmXET6.3 [123], and between XG and pectin fragments by barley HvXET3, HvXET4 and HvXET6 isoforms [125], indicates that plants have developed sophisticated and variable mechanisms to influence firmness, porosity, and flexibility of plant CWs [58], and thus the ability to modify the properties of CWs that otherwise would lack adaptability to the actual needs. These specific XET isoforms are expressed only in plants in a defined developmental stage or in a certain plant organ or tissue, or exclusively during the limited stages of plant development, as it can be gleaned from the data regarding the localisation of these enzymes based on their activities or gene expression [123,125,135,162,178,179,180]. The significance of these hetero-transglycosylation reactions by broad specific XET enzymes may be related to the structural roles of individual polysaccharides in PCW and SCW. The flexibility and dynamic properties of CWs that are required to fulfill multiple roles during plant growth and development result from complex interactions between the major CW polysaccharides in muro such as cellulose, (glucurono-arabino) xylans or GlcMan, MLG, XG and, pectin polysaccharides, structural proteins and polyphenolic compounds, inorganic molecules, and expansin proteins [125].

The fundamental complexities between the major structural polysaccharides in monocot and dicot plant CWs have been outlined [66,82,181,182,183], although the significance of homo- and hetero-transglycosylation reactions for the CW structure and function needs to be further explored, e.g., by mutant knockout plants lacking enzymes that catalyse these reactions. This multipronged research of XET enzymes is required to understand the fine structure, processes, and inter-connections between structural components of CWs, and how these relationships assist with better understanding the roles of XETs in plant cells from macro to atomic levels.

## Figures and Tables

**Table 1:** Substrate specificities and other properties of selected plant XET and XTH enzymes.

Enzyme	Plant Source	Enzyme Purity ^a^	Donor ^b^	Acceptor ^b^	Assay Method ^c^ Reference
HvXET3	*Hordeum vulgare* L.	+	XG, HEC	XG-OS, MLG-OS, Cello-OS, Pu-OS, AraXyl-OS, La-OS, Xyl-OS, GlcMan-OS, Ara-OS, [α(1-4)GalA*p*]_5_	R, F [125,144,154]
HvXET4	*Hordeum vulgare* L.	+	XG, HEC	XG-OS, MLG-OS, Cello-OS, Pu-OS, AraXyl-OS, La-OS, Xyl-OS, GlcMan-OS, Ara-OS, [α(1-4)GalA*p*]_5_	R, F [125,144,154]
HvXET5	*Hordeum vulgare* L.	+	XG, CMC, HEC, MLG	XG-OS, Cello-OS	R, F [58]
HvXET6	*Hordeum vulgare* L.	+	XG, CMC, HEC, MLG	XG-OS, MLG-OS, Cello-OS, Pu-OS, AraXyl-OS, La-OS, Xyl-OS, GlcMan-OS, Ara-OS, [α(1-4)GalA*p*]_5_	R, F [121,125,144,154]
TmXET6.3	*Tropaeolum majus* L.	+/−	XG, HEC	XG-OS, MLG-OS, Cello-OS, Pu-OS, AraXyl-OS, La-OS, Xyl-OS, GlcMan-OS, Ara-OS	F [123,159]
PttXET16A	*Populus tremulus x tremuloides* L.	+	XG	XG-OS	C [127,138]
PrXTH1	*Pinus radiata* L.	−	XG	XG-OS, Cello-OS	C [157]
EfXTH-A	*Equisetum fluviatile* L.	−	XG, cellulose, MLG	XG-OS	R [117]
EfXTH-H	*Equisetum fluviatile* L.	−	XG, cellulose, MLG	XG-OS	R [117]
EfXTH-I	*Equisetum fluviatile* L.	−	XG, cellulose, MLG	XG-OS	R [117]
EfHTG	*Equisetum fluviatile* L.	+/−	XG, cellulose, MLG	XG-OS	R, F [136]

^a^ Enzyme purity: (+) near-homogenous; (−) not demonstrated; (+/−) purity demonstrated for native but not for recombinant EfHTG and TmXET6.3, expressed in *Pichia*. ^b^ Abbreviations for donor and acceptor substrates defined in [123,125]. ^c^ Assay method: F—fluorimetric; C—colorimetric; R—radiometric.

## Supplementary Materials

The following are available online. Supplementary Data Set S1 contains the list of sequences of the GH16 family and their properties, such as tissue localisation and suggested function (sheet 1), and the FASTA alignment file (sheet 2) used for the generation of the unrooted phylogenetic tree (Figure 2C).

## Abbreviations

[α(1-4)GalAp]5, penta-galacturonic acid; Ara-OS, arabinan oligosaccharide(s)s; AraGal-OS, arabinogalactan oligosaccharide(s); AraXyl-OS, arabinoxylan oligosaccharide(s); AraXyl(s), arabinoxylan(s); CW(s), cell wall(s); CAZy, Carbohydrate-Active enZYmes; CSL, cellulose synthase-like; Cello-OS, cellooligosaccharide(s); CMC, carboxymethyl cellulose; DP(s), degree(s) of polymerisation; GalGlcMan; galactoglucomannan; GalMan(s), galactomannan(s); GalMan-OS, galactomannan oligosaccharide(s); GH(s), glycoside hydrolase(s); GlcMan-OS, glucomannan oligosaccharide(s); GlcMan(s), glucomannan(s); GT(s), glycosyl transferase(s); HEC, hydroxyethyl cellulose; HG, homogalacturonan; HTG, hetero-trans-β-glucanase; IUBMB, International Union of Biochemistry and Molecular Biology; IUPAC, International Union of Pure and Applied Chemistry; KEGG, Kyoto Encyclopaedia of Genes and Genomes; La-OS, laminarioligosaccharide(s); Man(s), mannan(s); Man-OS, mannan oligosaccharide(s); MLG(s), (1,3;1,4)-β-d-glucan(s) or mix-linkage glucans; MLG-OS, mix-linkage oligosaccharide(s); PCW(s), primary cell wall(s); PDB, Protein Data Bank; Pu-OS pustulan oligosaccharide(s); RGI/II, rhamnogalacturonan I/II; SCW(s), secondary cell wall(s); UniProtKB, Universal Protein KnowledgeBase; XEH(s), xyloglucan endohydrolase(s); XET(s), xyloglucan endo-transglycosylase(s); XG(s); xyloglucan(s); XG-OS, xyloglucan oligosaccharide(s); XTH, xyloglucan transglycosylase/hydrolase; Xyl(s), xylan(s); Xyl-OS, xylan oligosaccharide(s).
