# Supplementary material for: Plant Xyloglucan Xyloglucosyl Transferases and the Cell Wall Structure: Subtle but Significant
Source: Molecules. 2020 Nov 29;25(23):5619. doi: 10.3390/molecules25235619 (PMC7729885; doi:10.3390/molecules25235619)
Supplement: Supplementary file 1 [file molecules-25-05619-s001.pdf]

Supplementary Data Set S1. The list of sequences of the GH16 family and their properties, such as tissue localisation and suggested function.

Organism (plant/yeast)	Gene name	Accession	Tissue localisation	Suggested function	Reference	Entry name used in Figure 2C
Arabidopsis thaliana	XTH3	Q9LJR7	Predominantly expressed in flower buds	XET activity/ CW biogenesis	[121, 184]	Q9LJR7 ARATH
Arabidopsis thaliana	XTH31	P93046	Predominantly expressed in roots	XEH activity/ CW biogenesis	[184 - 187]	P93046 AtXTH31
Hordeum vulgare	HVXEA	P93671	Predominantly expressed in first leaf base	XET activity/ CW biogenesis	[124, 140, 152, 161]	P93671 HvXET3
Hordeum vulgare	HVXEB	P93672	Predominantly expressed in spike	XET activity/ CW biogenesis	[124, 140, 152, 161]	P93672 HvXET4
Hordeum vulgare	EXT	P93668	Protein isolated from 7-days-old seedlings	XET activity/ CW biogenesis	[97, 188]	P93668 HvXET5
Hordeum vulgare	XET6	B1P1S7	Predominantly expressed in flower at anthesis	XET activity/ CW biogenesis	[120, 124]	B1P1S7 HvXET6
Tropaeolum majus	N/A	Q07524	Expressed only in cotyledons	XEH activity/ CW biogenesis	[137, 159, 189]	Q07524 2UWA NXG1
Tropaeolum majus	N/A	2UWC	Expressed only in cotyledons	XEH activity/ CW biogenesis	[137, 189]	2UWC NXG2
Tropaeolum majus	XET1	Q41614	Predominantly expressed in young epicotyls and roots	XET activity/ CW biogenesis	[159]	Q41614 TmXET1
Tropaeolum majus	xet6.3	V5ZEF7	Protein found only in germinating seeds	XET activity/ CW biogenesis	[122]	V5ZEF7 TmXET6.3
Equisitum fluovatile	HTG	A0A0K2WA62	In shoots	HTG activity/ CW biogenesis	[134, 135]	A0A0K2WA62 EfHTG
Equisitum fluovatile	EfXTH-A	MT495433*	N/A	XET activity/ CW biogenesis	[116]	EfXTH-A
Equisitum fluovatile	EfXTH-H	MT495434*	N/A	XET activity/ CW biogenesis	[116]	EfXTH-H
Equisitum fluovatile	EfXTH-I	MT495435*	N/A	XET activity/ CW biogenesis	[116]	EfXTH-I
Populus tremulus x tremuloides	XET16A	Q8GZD5	In vascular tissues	XET activity/ CW biogenesis	[101, 126]	Q8GZD5 PttXET16A
Pinus radiata	XTH1	A0A059SVJ4	In stems (inclined)	XET activity/ CW biogenesis	[155, 190]	A0A059SVJ4 PrXET1
Saccharomyces cerevisiae	CRH1	P53301	Fungal CWs	Transfer of chitin to 1,6-β-glucan / CW biogenesis	[191 - 193]	P53301 CRH1
Saccharomyces cerevisiae	UTR2	P32623	Fungal CWs	Transfer of chitin to 1,6-β-glucan / CW biogenesis	[192 - 194]	P32623 CRH2

*GenBank nucleotide sequence accession number (BankIt2345959)

N/A, not available.

References

184. Yokoyama, R.; Nishitani, K. A comprehensive expression analysis of all members of a gene family encoding cell-wall enzymes allowed us to predict cis-regulatory regions involved in cell-wall construction in specific organs of Arabidopsis. Plant Cell Physiol. 2001, 42, 1025–1033, doi:10.1093/pcp/pce154.

185. Aubert, D.; Herzog, M. A new cDNA encoding a xyloglucan endo-transglycosylase-related polypeptide (AtXTR8) preferentially expressed in seedling, root and stem of Arabidopsis thaliana. Plant Sci. 1996, 121, 187–196, doi:10.1016/S0168-9452(96)04522-0.

186. Zhu, X.F.; Shi, Y.Z.; Lei, G.J.; Fry, S.C.; Zhang, B.C.; Zhou, Y.H.; Braam, J.; Jiang, T.; Xu, X.Y.; Mao, C.Z.; et al. XTH31, encoding an in vitro XEH/XET-active enzyme, regulates aluminum sensitivity by modulating in vivo XET action, cell wall xyloglucan content, and aluminum binding capacity in Arabidopsis. Plant Cell 2012, 24, 4731–4747, doi:10.1105/tpc.112.106039.

187. Kaewthai, N.; Gendre, D.; Eklof, J.M.; Ibatullin, F.M.; Ezcurra, I.; Bhalerao, R.P.; Brumer, H. Group III-A XTH genes of Arabidopsis encode predominant xyloglucan endohydrolases that are dispensable for normal growth. Plant Physiol. 2013, 161, 440–454, doi:10.1104/pp.112.207308.

188. Smith, R.C.; Matthews, P.R.; Schunmann, P.H.D.; Chandler, P.M. The regulation of leaf elongation and xyloglucan endotransglycosylase by gibberellin in "Himalaya" barley (Hordeum vulgare L.). J. Exp. Bot. 1996, 47, 1395–1404, doi:10.1093/jxb/47.9.1395.

189. de Silva, J.; Jarman, S.; Arrowsmith, D.A.; Stronach, M.S.; Chengappa, S.; Sidebottom, C.; Reid, J.S.G. Molecular characterization of a xyloglucan-specific endo-(1-4)-β-ᴅ-glucanase (xyloglucan endotransglycosylase) from nasturtium seeds. Plant J. 1993, 3, 701–711, doi:10.1046/j.1365-313X.1993.03050701.x.

190. Valenzuela, C.; Ramos, P.; Carrasco, C.; Moya-León, M.A.; Herrera, R. Cloning and characterization of a xyloglucan endo-transglycosylase/ hydrolase gene expressed in response to inclination in radiata pine seedlings. Tree Genet. Genomes 2014, 10, 1305–1315, doi:10.1007/ s11295-014-0762-9.

191. Arroyo, J.; Garcia-Gonzalez, M.; Garcia-Saez, M.I.; Sanchez-Perez, M.; Nombela, C. DNA sequence analysis of a 23,002 bp DNA fragment of the right arm of Saccharomyces cerevisiae chromosome VII. Yeast 1997, 13, 357–363, doi:10.1002/(SICI)1097-0061(19970330)13:4<357::AID-YEA77>3.0.CO;2-J.

192. Rodriguez-Peña, J.M.; Cid, V.J.; Arroyo, J.; Nombela, C. A novel family of cell wall-related proteins regulated differently during the yeast life cycle. Mol. Cell Biol. 2000, 20, 3245–3255, doi:10.1128/mcb.20.9.3245-3255.2000.

193. Cabib, E.; Blanco, N.; Grau, C.; Rodriguez-Pena, J.M.; Arroyo, J. Crh1p and Crh2p are required for the cross-linking of chitin to beta(1-6)glucan in the Saccharomyces cerevisiae cell wall. Mol. Microbiol. 2007, 63, 921–935, doi:10.1111/j.1365-2958.2006.05565.x.

194. Dietrich, F.S.; Mulligan, J.T.; Hennessy, K.M.; Yelton, M.A.; Allen, E.; Araujo, R.; Aviles, E.; Berno, A.; Brennan, T.; Carpenter, J.; et al. The nucleotide sequence of Saccharomyces cerevisiae chromosome V. Nature 1997
